# Chemical composition and the nutritive value of pistachio epicarp (*in situ* degradation and *in vitro* gas production techniques)

**Published:** 2014

**Authors:** Somayeh Bakhshizadeh, Akbar Taghizadeh, Hossein Janmohammadi, Sadegh Alijani

**Affiliations:** 1 *MSc student, Department of Animal Sciences, Faculty of Agriculture, University of Tabriz, Tabriz, Iran;*; 2 *Department of Animal Sciences, Faculty of Agriculture, University of Tabriz, Tabriz, Iran.*

**Keywords:** *In situ* degradation, *In vitro* gas production, Pistachio epicarp, Polyethylene glycol

## Abstract

The nutritive value of pistachio epicarp (PE) was evaluated by *in situ *and *in vitro* techniques. Chemical analysis indicated that PE was high in crude protein (11.30%) and low in neutral detergent fiber (26.20%). Total phenols, total tannins, condensed tannins and hydrolysable tannins contents in PE were 8.29%, 4.48%, 0.49% and 3.79%, respectively. Ruminal dry matter and crude protein degradation after 48 hr incubation were 75.21% and 82.52%, respectively. The gas production volume at 48 hr for PE was 122.47 mL g^-1^DM. As a whole, adding polyethylene glycol (PEG) to PE increased (*p* < 0.05) gas production volumes, organic matter digestibility and the metabolizable energy that illustrated inhibitory effect of phenolics on rumen microbial fermentation and the positive influence of PEG on digestion PE. The results showed that PE possessed potentials to being used as feed supplements.

## Introduction

Due to shortage of water and locally produced feeds in many developing and underdeveloped countries, livestock often suffer from under-feeding and malnutrition. Thus, better utilization of non-agricultural by-products which do not compete with human foods is imperative. One of the agro-industrial co-products is pistachio epicarp (PE) which offers some promises of overcoming this problem. In Iran, production pistachio by-products (PB) exceeds 400,000 tons per year.^1^ The nutritive value of PE varies depending on variations in the pistachios cultivars, growing practices, kernel maturity and the dehulling process. The crude protein (CP) content of PB varies from 92 to 120 g kg^-1 ^on a dry matter (DM) basis and the metabolizable energy (ME) content of PB may vary from 7.10 to 7.50 MJ kg^-1^ DM.^2^ However, PE have been reported to contain high levels of tannins in both hydrolysable and condensed forms.^3^ Therefore, the value of PE for ruminants is offset by their potentially negative effects on protein utilization, and the risk of toxicity when intake is high.^4^ Polyethylene glycol (PEG), a non-nutritive synthetic polymer, has a high affinity to tannins and can make them inert by forming tannin-PEG complexes.^5^ Polyethylene glycol can also liberate protein from tannin–protein complexes and/or prevent their formation.^6^ Therefore, PEG has been used to mitigate adverse effects of tannins on rumen fermentation.

The aim of the current study was to assess the chemical composition and nutritive value of PE using *in situ* and *in vitro* gas production techniques.

## Materials and Methods

**Pistachio epicarp. **Fresh PE (*Pistacia vera*) was collected from pistachio plant cleaning factories of Ghazvin, Iran. Samples were oven-dried at 50˚C for 48 hr and stored in sealed plastic bags. Samples were milled through a 2 mm screen for *in situ* analysis, *in vitro* gas production and chemical analysis.

**Chemical composition. **Pistachio epicarp dry matter (DM, Method ID 934.01), ash (Method ID 942.05), ether extract (EE, Method ID 920.30) and crude protein (Method ID 984.13) were determined by procedures of Association of Official Analytical Chemists (AOAC).^7^ The NDFand ADF concentrations were determined using the methods of Van Soest *et al*. without sodium sulphite.^8^ The NDF was analyzed without amylase with ash included. Total phenolics (TP) were measured using Folin Ciocalteau method according to Makkar *et al*. ^5^ Total tannin (TT) was determined after adding insoluble PVPP and reacting with Folin Ciocalteau reagent.^9^ Tannic acid was used to express TP and TT. The condensed tannins (CT) were measured by the HCL-butanol method.^12^ Hydrolysable tannins were analyzed using the Rhodanine assay,^9^ and results are expressed as gallotannin.

***In situ*** **DM and protein degradability analysis. **Rumen degradation characteristics of feeds were calculated after the incubation of 5 g sample of PE in nylon bags.^10^ The bag size was 12 cm × 6 cm with a pore size of 50 µm. There were four bags for each incubation time. Bags were incubated at 0, 2, 4, 6, 8, 12, 16, 24, 36, 48, 72, and 96 hr. The sheep equipped with ruminal fistulae that were fed daily containing of 400 g lucerne, 300 g barley and 300 g soybean meal. In each sheep two bags were used for each time interval. Animals had free access to water. After removal, bags were washed under tap water for at least 15 min until the washing water was clear. After hand squeezing, bags were dried for 48 hr at 105 ˚C then weighed. Feed residues were recovered from each bag and stored pending analysis for Kjeldahl nitrogen. The value of degradability at time 0 was obtained by washing four bags under tap water for at least 15 min. For each bag, the residue was analyzed for DM and nitrogen. The percentage of degradability (Y) of DM and nitrogen at time (t) was obtained from an exponential curve of the type: 

Y = a + b (1 − e^-ct^)

which was fitted to the experimental data by iterative regression analysis.^11^ In this equation, e is the base of natural logarithm, the constant ‘a’ represents the soluble and very rapidly degradable component and ‘b’ represents the insoluble but potentially degradable component, which degrades at a constant fractional rate (c) per time. The effective degradability of DM and protein for PE were then estimated by the following equation: 

Effective degradability (g kg^-1^ DM) = a + bc / (c + k)

where k refers to the fractional outflow rate of small particles from rumen. A value of 0.04 per hr for k was used.

***In vitro*** **gas production analysis. **Rumen fluid for the *in vitro* digestibility tannin bioassay was obtained from two healthy mature Gezel wether sheep of with live weight 34.00 kg (±1.50) were fed a daily ration 400 g lucerne, 300 g barley and 300 g soybean meal twice daily at 09:00 and 17:00 hours.

Gas production was completed as described by Menke and Steingass.^12^ About 300 mg dried PE alone was incubated in duplicate with 20 mL rumen liquor- artificial saliva mixture (1:2) in calibrated glass syringes at 39 ˚C. A second assay was carried out with 300 mg PE incubated with 600 mg polyethylene glycol (PEG 6000) as described by Makkar *et al*.^5^ Readings were recorded after 2, 4, 6, 8, 12, 16, 24, 36, 48, 72, and 96 hr of incubation and cumulative gas production data was fitted into an exponential equation as described in previous section.^11^

Feed OMD (g kg^-1^ DM) and metabolizable energy (ME) in MJ kg^-1^ DM were estimated by equations of Menke and Steingass,^12^ based on 24 hr gas production (Gas, mL) and CP content (g kg^-1^DM) as: 

OMD (g kg^-1 ^DM) = 148.8 + 8.89 Gas + 4.5 CP + 0. 651 XA

and 

ME (MJ kg^-1^ DM) = 2.20 + 0.136 Gas + 0.057 CP + 0.0029 CP

where OMD is OM digestibility, ME is metabolizable energy; CP is crude protein in g per 100 g DM; XA is ash in g per 100 g DM; and GAS is the net gas production (mL).^2^ Net gas production data were converted from 300 to 200 mg after 24 hr of incubation.

**Statistical analysis. **Data obtained from *in situ* and *in vitro* gas production was subjected to ANOVA as a completely randomized design with four replicates by the GLM procedure, and treatment means were compared by the Duncan test. The relationship between *in situ *CP degradability (ISCPD) with *in situ* dry matter degradability (ISDM), determined by linear regression using the PROC REG procedure of SAS (Version 8.2; SAS Institute, Carry, USA). 

## Results

**Chemical composition. **The chemical composition of PE is presented in Table 1. Crude protein content in PE was 11.30% that indicated PE was high in CP, also the results showed that PE was low in NDF content. 

**Fig. 1:**
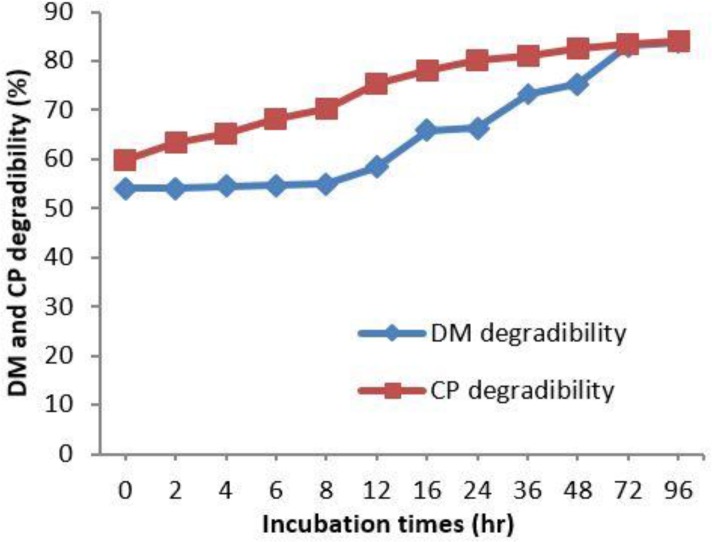
Pattern of in situ CP and DM degradability of PE in incubation times

***In situ*** **DM and protein degradability.** Figure 1 shows the pattern of *in situ* CP and DM degradability of PE at incubation times. The disappearance of DM and CP increased with increasing time of incubation. After 96 hr of incubation, the CP and DM degradability for PE were 84.14% and 85.83%, respectively.

**Fig. 2:**
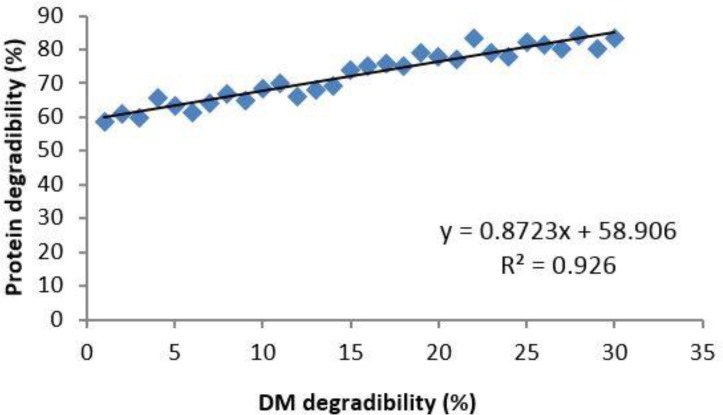
Correlation between 48 hr DM *in situ *degradability and 48 hr protein degradability data from Pistachio epicarp

Characteristics of the DM and CP *in situ* degradation of PE are given in Table 2. Soluble DM and CP (rapidly degraded fraction) for PE stood at 52.45% and 59.56%, respectively. Slowly degraded fraction of DM and CP were 40.11% and 23.55%, respectively. The relationship between *in situ* DM degradability and CP degradability is shown in Figure 2. There is a high relationship (r^2^ = 0.926; *p* < 0.05) in all incubation times.

**Fig. 3:**
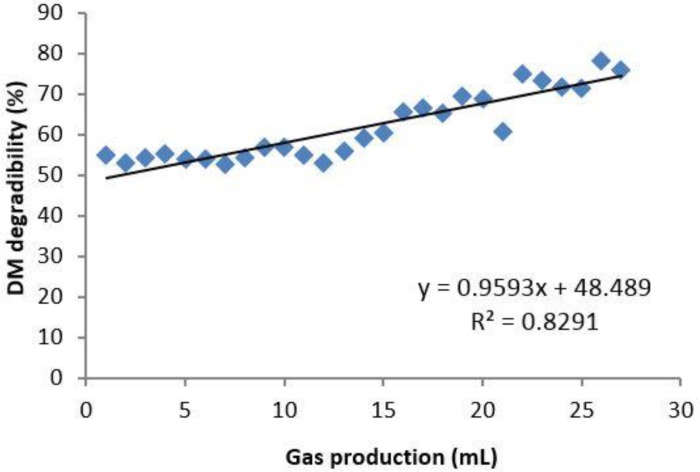
Correlation between 48 hr *in vitro* gas production and 48 hr DM *in situ* degradability from Pistachio epicarp

**Table 1:** Chemical composition of pistachio epicarp (g kg^-1^DM).

**Sample**	**DM (g kg** ^-1^ **)**	**CP (%)**	**ADF (%)**	**NDF (%)**	**EE (%)**	**Ash (%)**	**TP (%)**	**TT (%)**	**CT (%)**	**HT (%)**
**Pistachio epicarp**	223.3	113.00	187.00	262.00	78.00	150.80	82.90	44.80	4.90	37.90

DM: dry matter (g kg^-1^fresh weight); CP: crude protein; ADF: acid detergent fiber; NDF: neutral detergent fiber; EE: ether extract; TP: total phenols; TT: total tannins as tannic acid equivalents; CT: condensed tannins; HT: hydrolysable tannins.

**Table 2:** Degradation parameters of DM and CP in pistachio epicarp

**Items**	**Parameters**				
	**a**	**b**	**c**	**ED**	**RSD**
** ISDM**	52.45	40.11	0.01	72.00	1.89
** ISCPD**	59.56	23.55	0.07	78.30	0.59

ISDM*: in* *situ* dry matter degradability; ISCPD: *in* *situ* CP degradability, a = rapidly degraded fraction (%), b = slowly degraded fraction (%), c = rate of degradation (fraction per hr), RSD = residual standard deviation. ED = effective degradability with passage rate of 0.02 per hr.

**Table 3:** *In vitro* gas production and estimated parameters pistachio epicarp

	**Incubation times (hr)**											
	**2**	**4**	**6**	**8**		**12**	**16**	**24**	**36**	**48**	**72**	**96**
**Control**	20.16^b^	21.78^b^	22.76^b^	25.08^b^		34.82^b^	65.91^b^	96.99^b^	113.92^b^	122.47^b^	131.86^b^	142.7^b^
**PEG**	24.80^a^	28.33^a^	29.87^a^	32.13^a^		70.60^a^	109.80^a^	135.27^a^	163.53^a^	181.63^a^	195.70^a^	204.23^a^
**SEM**	0.26	0.78	0.0785	0.064		1.59	2.98	3.65	5.94	5.74	6.11	6.12
	**Estimated parameters**											
	**a+b ** **(mL g-1 DM)**				**c ** **(mL per hr)**				**ME ** **(MJ kg-1 DM)**		**OMD** **(g kg-1 DM)**	
**Control**	151.70^b^				0.03 b				5.86 b		38.20 b	
**PEG**	227.07^a^				0.04 a				6.90 a		45.00 a	
**SEM**	0.37				0.00				0.07		0.43	

ab Similar superscripts indicate non-significant differences in each row (*p* > 0.05). PEG: Polyethylene glycol. a + b: Potential gas production*; *c: Rate constant of gas production during incubation; ME: Metabolizable energy; OMD: organic matter digestibility; SEM: Standard error means.

***In vitro*** **gas production.** The cumulative gas production is shown in Table 3. After 96 hr of incubation volume of produced gas in PE was 142.70 mL g^-1^DM. The addition of PEG increased gas production at all times of incubation (Table 3). Kinetics of gas production (a+b and c), ME and OMD were also increased by PEG incorporation (*p* < 0.05).

## Discussion

The CP and TP contents of PE were similar to that reported by Mahdavi *et al*. while the NDF and ADF contents were lower than that reported by these authors.^3^ The amount of total phenol and total tannins for PB (9.13% and 7.90%, respectively) reported by Bagheripour *et al*. were higher than obtained values ​​for PE in this study.^1^ Generally, differences can be depended on the maturity stage, species or variety,^13^ drying methods, the condition environment,^14^ and soil type.^15^ Nitrogen, NDF and phenolic concentration suggest that PE have the potential to be used as feed supplements.

Mahdavi *et al*. showed The values of a, b and c for DM dried PE were 44.69%, 44.31% and 0.04%, respectively.^3^ The DM degradabilities of PE at 48 hr were higher than those reported by Mahdavi *et al*.^3^ Variation in protein degradability is believed to be related to the proportion of structural and non-structural protein and carbohydrate fractions, which in turn affects their solubility and bio-availability.^16^

Bagheripour *et al*. reported the gas production of PB at 24 hr of incubation about 35.00 mL g^-1 ^OM which was lower than the value reported for PE in this study.^1^ Tacon *et al*.^16^ reported that OMD feeds and gas production was reduced with increasing of phenolic contents. The phenolic contents in study of Bagheripour *et al*. were higher than the values ​​for PE in this study (9.13% vs 8.29). ^1^

Inclusion of PEG increased the gas production in PE, the increasing in gas production at 24 hr incubation was about 39.46%. The similar results have been reported by Bagheripour *et al*. who found PEG utilization increased gas production in PB about 14%.^1^

Increased gas production and OMD due to addition of PEG *in vitro* suggests a negative influence of tannins on digestibility.^17^ Inactivation of tannins through PEG binding increases availability of nutrients resulting in increased microbial activity and gas production. Similar results were obtained by Khazaal *et al*. when using PEG as the phenolic binding agent in conjunction with an in vitro gas technique for assessment of secondary compounds in browse.^18^

There was a positive correlation between 48 hr gas production and 24 hr DMD data (*p* < 0.05, r^2 ^=0.82), (Fig. 3). This is in agreement with finding of Rodrigues *et al*.^19^ but not with that of Blummel and Ørskov,^20^ who did not find any correlation between these parameters in barley and wheat straws. Kamalak *et al*. suggested that the relationship between these parameters varies with type of forage.^21^ The positive and significant (*p* < 0.05) correlation between *in situ* degradability and *in vitro* gas production data suggests that either method could be used to estimate nutritive value of such feeds. Previous experiments have shown a positive relationship between *in situ* degradability and both voluntary intake and *in vivo* digestibility^10^^,^^22^ as well as 24 hr *in vitro* gas production and metabolizable energy for ruminants from various forages.^23^ Khazaal* et al*. reported that *in situ* method should be used with caution when estimating the nutritive value of high phenolic feeds.^24^ The potential negative effect of phenolic compounds on rumen microbial fermentation is unlikely to be detected by *in situ* method. In this respect in vitro methods are more reliable in detecting inhibitory compounds in feeds. The *in vitro* gas production technique is a closed system with limited supply of rumen liquor where if there is any anti-nutritive compound, it is likely to affect the activity of rumen microbes. 

 In conclusion, our results showed that the PE evaluated in the present study can be used for supplying part of protein requirements. The *in situ *degradability data suggest that PE have a high nutritive value. *In vitro* gas production of PE was increased when that combined with PEG. In order to further optimize PE use as a feed supplement, more in vivo studies are warranted.
